# Long-term cost-effectiveness of interventions for loss of electricity/industry compared to artificial general intelligence safety

**DOI:** 10.1186/s40309-021-00178-z

**Published:** 2021-09-20

**Authors:** David Denkenberger, Anders Sandberg, Ross John Tieman, Joshua M. Pearce

**Affiliations:** 1 Alliance to Feed the Earth in Disasters (ALLFED), Fairbanks, AK 99775 USA; 2 University of Alaska Fairbanks, Fairbanks, AK 99775 USA; 3 Future of Humanity Institute, University of Oxford, Oxford, UK; 4 Thompson Centre for Engineering Leadership & Innovation, Ivey Business School, Department of Electrical & Computer Engineering, Western University, Canada, USA

**Keywords:** Cost-effectiveness analysis, Global catastrophic risk, Existential risk, Artificial intelligence, Solar storm, Nuclear weapons

## Abstract

Extreme solar storms, high-altitude electromagnetic pulses, and coordinated cyber attacks could disrupt regional/global electricity. Since electricity basically drives industry, industrial civilization could collapse without it. This could cause anthropological civilization (cities) to collapse, from which humanity might not recover, having long-term consequences. Previous work analyzed technical solutions to save nearly everyone despite industrial loss globally, including transition to animals powering farming and transportation. The present work estimates cost-effectiveness for the long-term future with a Monte Carlo (probabilistic) model. Model 1, partly based on a poll of Effective Altruism conference participants, finds a confidence that industrial loss preparation is more cost-effective than artificial general intelligence safety of ~ 88% and ~ 99+% for the 30 millionth dollar spent on industrial loss interventions and the margin now, respectively. Model 2 populated by one of the authors produces ~ 50% and ~ 99% confidence, respectively. These confidences are likely to be reduced by model and theory uncertainty, but the conclusion of industrial loss interventions being more cost-effective was robust to changing the most important 4–7 variables simultaneously to their pessimistic ends. Both cause areas save expected lives cheaply in the present generation and funding to preparation for industrial loss is particularly urgent.

## Introduction

Civilization relies on a network of highly interdependent critical infrastructure (CI) to provide basic necessities (water, food, shelter, basic goods), as well as complex items (computers, cars, space shuttles) and services (the internet, cloud computing, global supply chains), henceforth referred to as industry. Electricity and the electrical infrastructure that distributes it plays an important role within industry, providing a convenient means to distribute energy able to be converted into various forms of useful work. Electricity is one component of industry albeit a critical one. Industry provides the means to sustain advanced civilization structures and the citizens that inhabit them. These structures play a critical role in realizing various futures by allowing humanity to discover and utilize new resources, adapt to various environments, and resist natural stressors.

Though industry is capable of resisting small stressors, a sufficiently large event can precipitate cascading failure of CI systems, resulting in a collapse of industry. If one does not temporally discount the value of future people, the long-term future (thousands, millions, or even billions of years) could contain an astronomically large amount of value [18]. Events capable of curtailing the potential of civilization (existential risks, such as human extinction or an unrecoverable collapse) would prevent such futures from being achieved, implying reducing the likelihood of such events is of the utmost importance [100]. Reducing the prevalence of existential risks factors; events, systemic structures, or biases which increase the likelihood of extinction but do not cause extinction by themselves is also highly valuable. Complete collapse or degraded function of industry would drastically reduce humanity’s capacity to coordinate and deploy technology to prevent existential risks, representing an existential risk factor. Consequently, interventions preventing loss of industry, reducing the magnitude of impacts, or increasing speed of recovery could be extremely valuable.

Existential risk research is, by nature, future focused, requiring the investigation of events that have not yet occurred. Futures studies methodologies are often applied to uncover salient trends or events, and explore potential causal structures [54, 123]. Probabilistic modeling techniques can then be used to determine the likelihood of such events occurring, including adequate treatment of uncertainty [101]. The cost-effectiveness modeling approach outlined in this paper is an example of this, attempting to assess the marginal utility of losing industry interventions on improving the long-term future. This approach could guide future efforts to assess the relative cost-effectiveness of interventions for different risks, existential or otherwise. More practically, this research can inform prioritization efforts of industrialized countries by providing estimates of the cost of global industrial collapse, and the utility of resilience interventions. This is relevant to the European Union which has a highly industrialized economy, providing $2.3 Trillion USD of the $13.7 Trillion USD global total of value add manufacturing [122]. The EU has shifted toward a more proactive foresight approach about natural and man-made disasters, noting the importance of rare high-impact events, systemic risks, and converging trends requiring better data and forecasting to drive a more ambitious crisis management system [47]. Still, it is clear that most academic and institutional emphasis has been on “ordinary” rather than extreme disasters, and risks from industry to the public and environment rather than widespread failures of industrial services causing harm.

The integrated nature of the electric grid, which is based on centralized generation makes the entire system vulnerable to disruption.^1^ There are a number of anthropogenic and natural catastrophes that could result in regional-scale electrical grid failure, which would be expected to halt the majority of industries and machines in that area. A high-altitude electromagnetic pulse (HEMP) caused by a nuclear weapon could disable electricity over part of a continent [16, 48, 66, 93]. This could destroy the majority of electrical grid infrastructure, and as fossil fuel extraction and industry is reliant on electricity [49], industry would be disabled. Similarly, solar storms have destroyed electrical transformers connected to long transmission lines in the past [117]. The Carrington event in 1859 damaged telegraph lines, which was the only electrical infrastructure in existence at the time. It also caused Aurora Borealis that was visible in Cuba and Jamaica [70]. This could potentially disable electrical systems at high latitudes, which could represent 10% of electricity/industry globally. Though solar storms may last less than the 12 h that would be required to expose the entire earth with direct line of sight, the earth’s magnetic field lines redirect the storm to affect the opposite side of the earth [117].

Lastly, both physical [6, 8, 69, 89, 111] and cyber attacks [3, 63, 90, 96, 118, 128, 130] could also compromise electric grids. Physical attacks include traditional acts of terrorism such as bombing or sabotage [130] in addition to EMP attacks. Significant actors could scale up physical attacks, for example by using drones. A scenario could include terrorist groups hindering individual power plants [126], while a large adversary could undertake a similar operation physically to all plants and electrical grids in a region.

Unfortunately, the traditional power grid infrastructure is simply incapable of withstanding intentional physical attacks [91]. Damage to the electric grid resulting in physical attack could be long lasting, as most traditional power plants operate with large transformers that are difficult to move and source. Custom rebuilt transformers require time for replacement ranging from months and even up to years [91]. For example, a relatively mild 2013 sniper attack on California’s Pacific Gas and Electric (PG&E) substation, which injured no one directly, was able to disable 17 transformers supplying power to Silicon Valley. Repairs and improvements cost PG&E roughly $100 million and lasted about a month [10, 102]. A coordinated attack with relatively simple technology (e.g., guns) could cause a regional electricity disruption.

However, a high-tech attack could be even further widespread. The Pentagon reports spending roughly $100 million to repair cyber-related damages to the electric grid in 2009 [57]. There is also evidence that a computer virus caused an electrical outage in the Ukraine [56]. Unlike simplistic physical attacks, cyber attackers are capable of penetrating critical electric infrastructure from remote regions of the world, needing only communication pathways (e.g., the Internet or infected memory sticks) to install malware into the control systems of the electric power grid. For example, Stuxnet was a computer worm that destroyed Iranian centrifuges [73] to disable their nuclear industry. Many efforts are underway to harden the grid from such attacks [51, 63]. The U.S. Department of Homeland Security responded to ~ 200 cyber incidents in 2012 and 41% involved the electrical grid [103]. Nations routinely have made attempts to map current critical infrastructure for future navigation and control of the U.S. electrical system [57].

The electric grid in general is growing increasingly dependent upon the Internet and other network connections for data communication and monitoring systems [17, 112, 118, 127, 135]. Although this conveniently allows electrical suppliers management of systems, it increases the susceptibility of the grid to cyber-attack, through denial of webpage services to consumers, disruption to supervisory control and data acquisition (SCADA) operating systems, or sustained widespread power outages [3, 72, 118, 120]. Thus global or regional loss of the Internet could have similar implications.

A less obvious potential cause is a pandemic that disrupts global trade. Countries may ban trade for fear of the disease entering their country, but many countries are dependent on imports for the functioning of their industry. If the region over which electricity is disrupted had significant agricultural production, the catastrophe could be accompanied by a ~ 10% food production shortfall as well. It is uncertain whether countries outside the affected region would help the affected countries, do nothing, or conquer the affected countries.

Larger versions of these catastrophes could disrupt electricity/industry globally. For instance, it is possible that multiple HEMPs could be detonated around the world, due to a world nuclear war [105] or due to terrorists gaining control of nuclear weapons. There is evidence that, in the last 2000 years, two solar storms occurred that were much stronger than the Carrington event [85]. Therefore, it is possible that an extreme solar storm could disable electricity and therefore industry globally. It is conceivable that a coordinated cyber or physical attack (or a combination) on many electric grids could also disrupt industry globally. Many of the techniques to harden the electric grid could help with this vulnerability as well as moving to more distributed generation and microgrids [23, 29, 75, 76, 103, 114]. An extreme pandemic could cause enough people to not show up to work such that industrial functioning could not be maintained. Though this could be mitigated by directing military personnel to fill vacant positions, if the pandemic were severe enough, it could be rational to retreat from high human contact industrial civilization in order to limit disease mortality.

The global loss of electricity could even be self-inflicted as a way of stopping rogue artificial general intelligence (AGI) [124]. As the current high agricultural productivity depends on industry (e.g., for fertilizers), it has been assumed that there would be mass starvation in these scenarios [107].

Repairing these systems and re-establishing electrical infrastructure would be a goal of the long term and work should ideally start on it immediately after a catastrophe. However, human needs would need to be met immediately (and continually) and since there is only a few months of stored food, it would likely run out before industry is restored with the current state of preparedness. In some of the less challenging scenarios, it may be possible to continue running some machines on the fossil fuels that had previously been brought to the surface or from the use microgrids or shielded electrical systems. In addition, it may be feasible to run some machines on gasified wood [31]. However, in the worst-case scenario, all unshielded electronics would be destroyed.

The aim of this paper is to assess the relative cost-effectiveness of preventing loss of industry as compared to AGI safety research. Results will inform global and regional decision makers (government, business) in prioritization of resilience interventions for industrial collapse. AGI was selected due to being considered the greatest existential risk [100], receiving the largest amount of resources of any existential risk from philanthropic organizations associated with the existential risk community^2^ (though overall, biosecurity receives more resources) [77, 97, 132]. A favorable cost-effectiveness compared to AGI safety would make a strong case for loss of industrial civilization to be considered in the portfolio of existential risk reduction interventions. Also, the units of cost-effectiveness from the perspective of the long-term future are not intuitive, so a comparison to another cause makes the results more understandable. This paper focuses on catastrophes that only disrupt electricity/industry, rather than catastrophes that could disable industry and obscure the sun [39] or catastrophes that only obscure the sun (or affect crops directly in other ways) [33], analyzing the cost-effectiveness of interventions from a long-term perspective. First, this study will review interventions to both avoid a loss of electricity, but also to feed everyone with this loss. Then the benefits of AGI safety on the long term future will be reviewed and quantified. Next, two loss of industry interventions submodels are developed. The cost for an intervention based on alternative communication is also estimated. The development of a mathematical model also provides value by formalizing a causal structure and underlying assumptions concerning relevant phenomena, uncovering potential relations and dependencies between the two existential risks and facilitating further discourse in the literature.

## Background

### Review of potential solutions

An obvious intervention for HEMP is preventing a nuclear exchange, which would be the best outcome. However, it is not neglected, as it has been worked on for many decades [13, 32, 36, 37, 64, 84, 125] and is currently funded at billions of dollars per year quality adjusted [84, 85]. Other obvious interventions for HEMP that would also work for solar storms, and coordinated physical or cyber threats would be hardening the electrical grid against these threats. However, hardening just the U.S. electrical grid against solar storm and HEMP would cost roughly $20 billion [104]. Therefore globally, just from these two threats, it would be around $100 billion. Furthermore, adding hardening to cyber threats would be even more expensive. Again, preventing the collapse of electricity/industry would be the preferable option, but given the high cost, it may not happen. Even if it occurs eventually, it would still be preferable to have a backup plan in the near term and in the case that hardening is unsuccessful at stopping loss of industry.

A significant problem in loss of industry catastrophes is that of food supply [28]. One intervention is storing years worth of food, but it is too expensive to have competitive cost-effectiveness (and it would take many years so it would not protect humanity right away, and it would exacerbate current malnutrition) [15]. Furthermore, if electricity/industry is disabled for many years, food storage would be impractical. Stockpiling of industrial goods could be another intervention, but again it would be much more expensive than the interventions considered here.

Interventions for food production given the loss of industry include burning wood from landfills to provide fertilizer and high use of nitrogen fixing crops including legumes (peas, beans, peanuts, etc.) [28]. Also, nonindustrial pest control could be used. Despite pre-industrial agricultural productivity (~ 1.3 dry tons per hectare per year) [28], this could feed everyone globally. However, not everyone would be nearby the food sources, and losing industry would severely hamper transportation capability. Solutions for this problem include backup plans for producing more food locally, including expanding planted area (while minimizing impact to biodiversity, e.g., by expanding into the boreal forest/tundra enhanced by the nutrients from tree decomposition/combustion) and favoring high calorie per hectare foods such as potatoes, yams, sweet potatoes, lentils, and groundnuts [95]. Though clearing large areas of forest with hand saws would not be practical, it is possible to girdle the trees (remove a strip of bark around the circumference), let the trees dry out, and burn them. This has the advantage of releasing fertilizer to the soils. Another option involves producing “alternative foods,” which were proposed for sun-blocking catastrophes [32]. Some of these alternative foods would require industry, but producing non-industrial lower cost ones such as extracting calories from leaves [43] could be feasible. For transporting the food and other goods, ships could be modified to be wind powered and animals could pull vehicles [1]. A global network of shortwave radio transmitters and receivers would facilitate disseminating the message that there is a plan and people need not panic, and also allow for continuing coordination globally (see below).

Current awareness of interventions given loss of electricity/industry (hereafter “interventions”) is very low, likely in the thousands of people. Also, many of the interventions are theoretical only and need to be tested experimentally. There may be a significant amount of shortwave radio systems that are shielded from HEMP and have shielded backup power systems, but likely some addition to this capacity would be beneficial. This paper analyzes the cost-effectiveness of interventions from a long term perspective. It is unlikely that the loss of industry would directly cause human extinction. However, by definition, there would be a loss of industrial civilization for the global catastrophes. Furthermore, there could be a loss of anthropological civilization (basically cities or cooperation outside the clan). One definition of the collapse of civilization involves short-term focus, loss of long distance trade, widespread conflict, and collapse of government [27]. Reasons that civilization might not recover include (i) easily accessible fossil fuels and minerals are exhausted [88] (though there would be minerals in landfills), (ii) the future climate might not be as stable as it has been for the last 10,000 years [58], or (iii) technological and economic data and information might be lost permanently because of the trauma and genetic selection of the catastrophe [19]. If the loss of civilization were prolonged, a natural catastrophe, such as a super volcanic eruption or an asteroid/comet impact, could cause the extinction of humanity. Another way to far future impact is the trauma associated with the catastrophe making future catastrophes more likely, e.g., global totalitarianism [21]. A further route is worse values caused by the catastrophe could be locked in by artificial general intelligence (AGI) [20], though with the loss of industrial civilization, the advent of AGI would be significantly delayed, so the bad values could have decayed out by then.

#### Hypothetical scenario: severe pandemic-induced loss of industry

**Fig. 1:**
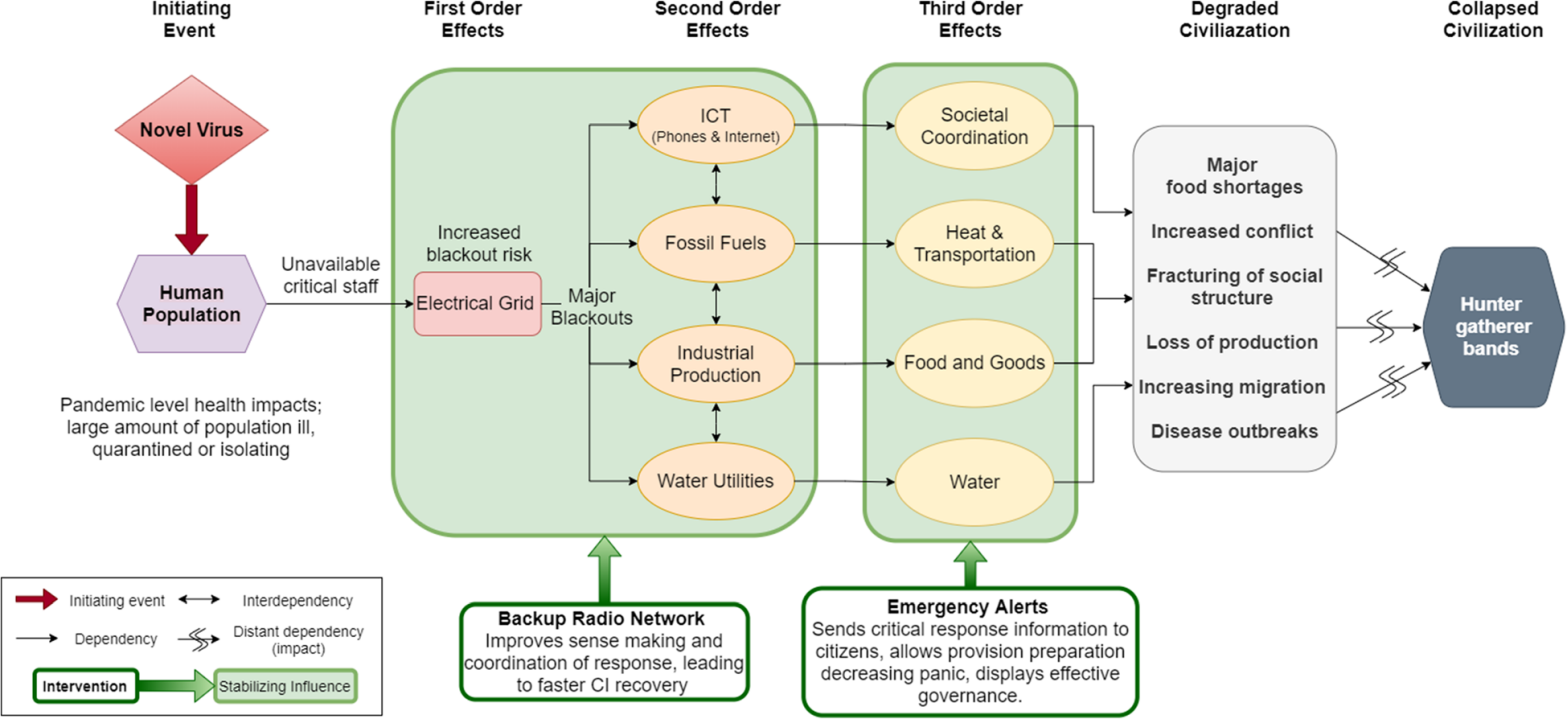
Causal diagram showing how a novel virus causing a pandemic could cause failure of critical infrastructure leading to loss of industrial civilization. The stabilizing influence of two potential interventions is highlighted in green

A severe pandemic represents a potential initial stressor capable of causing cascading failures through CI systems; these failures can be tightly dependent (< 24 h) or loosely dependent (taking days or months). As a result of the increasing number of infections from a novel pathogen, there could be a sharp decline in CI staff availability to work due to illness, fear, quarantine, or death. Over a period of weeks, electricity utilities could experience several small blackouts being unable to repair storm impacted grids components quickly. Unbalanced power supply and demand could eventually trigger power station shutoff protocols, and the power plants would not be able to be restarted due to insufficient staff, precipitating a large blackout. In turn, electricity-dependent infrastructure, such as ICT (internet, phone), water utilities, and fossil fuel (oil and gas) extraction would fail, which in turn would precipitate failure of logistics and distribution required to obtain replacement parts for the grid repair. Without preparation, people would be unable to obtain food and water in urban areas, and civil unrest and mass migrations would undermine social cohesion, reduce coordination, and cause splintering into smaller and smaller groups, precipitating the collapse of large-scale civilization (Fig. 1). (See Appendix 1 for extended qualitative case study).

Several potential low-cost interventions exist in the pandemic case: a government/social media/search engine message could be sent out once small power failures occurred informing citizens larger power outages are likely within 2 days and advising storing water and minimizing food consumption, for the large power outages. When a large outage occurs, a backup radio network (see Appendix 2 for details) could ascertain the magnitude of the outage (local, regional, global) and anticipate the severity of cascading effects. For severe events, e.g., a major grid failure leading to drastically reduced fuel production capacity, this network could be utilized to initiate predetermined response plans, such as relocation of urban citizens to rural areas to relieve pressure on food system logistics and provide assistance in harvesting of crops in the event fuel availability further diminishes mechanized agricultural production.

### Artificial general intelligence

AGI itself represents a major, independent risk. The artificial intelligence available now is narrow AI, i.e., it can generally only do a specific task, such as playing Jeopardy! [113]. However, there are concerns that as AI systems become more advanced, AGI will eventually be achieved [20]. Since AGI could perform all human tasks as well as or better than humans, this would include reprogramming the AGI. This would enable recursive self-improvement, so there could be an intelligence explosion [55]. Since the goals of the AGI may not be aligned with human interests [20] and could be pursued with great power, this implies a potentially serious risk [55]. AGI safety is a top priority in the existential risk community that seeks to improve humanity’s long term future [125]. Though there is uncertainty in when and how AGI may be developed, there are concrete actions that can be taken now to increase the probability of a good outcome [9].

We seek to compare the cost-effectiveness of losing industry interventions with AGI safety to discover whether these interventions should also be a top priority. Comparisons to other risks, such as asteroids [83], climate change [61], and pandemics [86], are possible, though these are generally regarded by the existential risk community as lower priority and therefore less informative.

## Methods

Given the large uncertainties in input parameters, cost-effectiveness was modeled using probabilistic sampling methods, producing a probability distribution of cost-effectiveness. Probabilistic uncertainty analysis is used widely in insurance, decision-support, and cost-effectiveness modeling [50]. In these models, uncertain parameters are represented by samples drawn from defined distributions that are combined into output samples that form a resultant distribution. Sampling methods used were: Monte Carlo (MC) in which N randomly selected points are independently drawn from input distributions; and Median Latin Hypercube (MLH) in which probability distributions are divided into M equiprobable intervals, independently shuffled and selected, with the median of the interval providing the sample point.

MC and MLH were selected because the probability distributions for various parameters do not come in a form that provides analytically tractable combinations. It also allows exploring parameter sensitivity.

The open source software called Guesstimate^3^ was originally used to implement the models, and they are available online (Guesstimate uses MC sampling). However, to enable more powerful analysis and plotting, the models were also implemented on the software Analytica 5.2.9. Analytica combines the uncertainties in all the inputs utilizing a Median Latin Hypercube analysis (similar to Monte Carlo, but better performing [67]) with the maximum uncertainty sample of 32,000 (run time on a personal computer was seconds). The results from the two software agreed within uncertainties due to the finite number of samples, giving greater confidence in the results.

The models consist of a loss of industry submodel estimating the risk and mitigation costs of industrial loss, and an AGI risk submodel estimating risk and mitigation costs of AGI scenarios. These two submodels then allow us to estimate the ratio and confidence of cost-effectiveness.

**Fig. 2:**
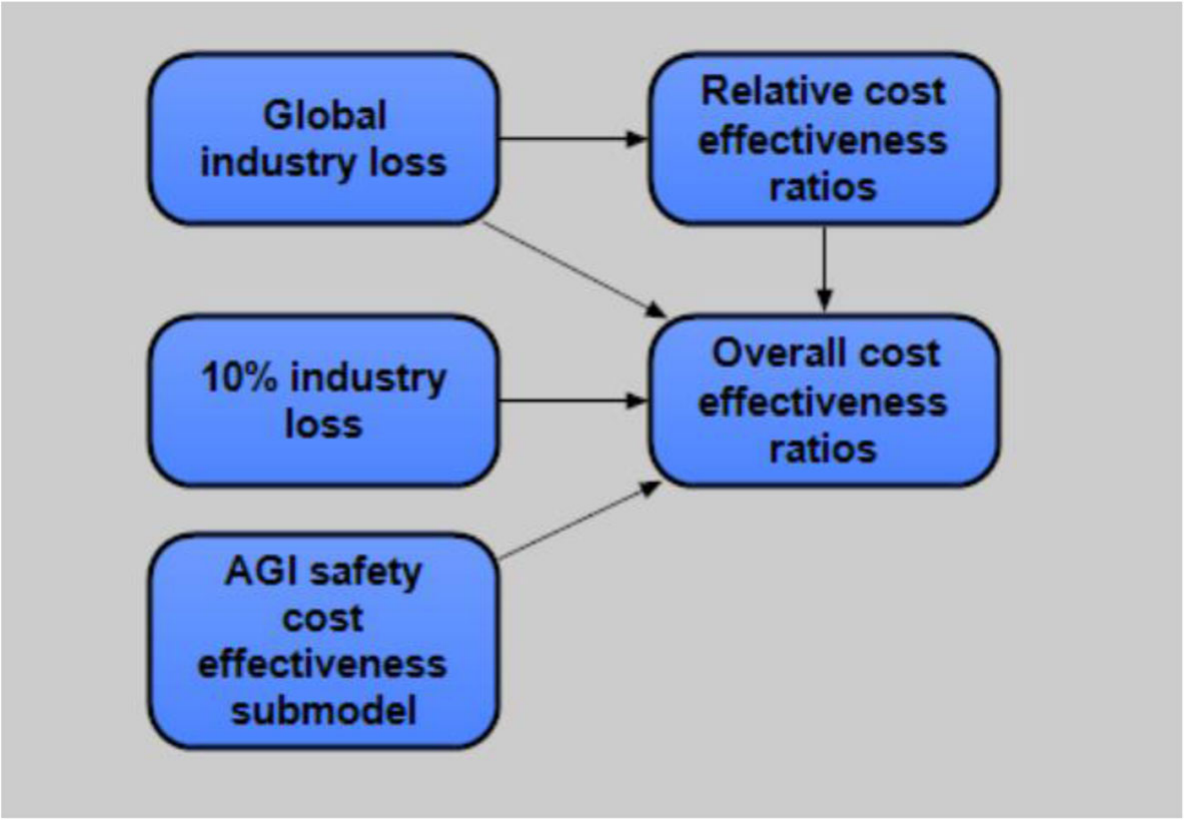
Model overview

**Fig. 3:**
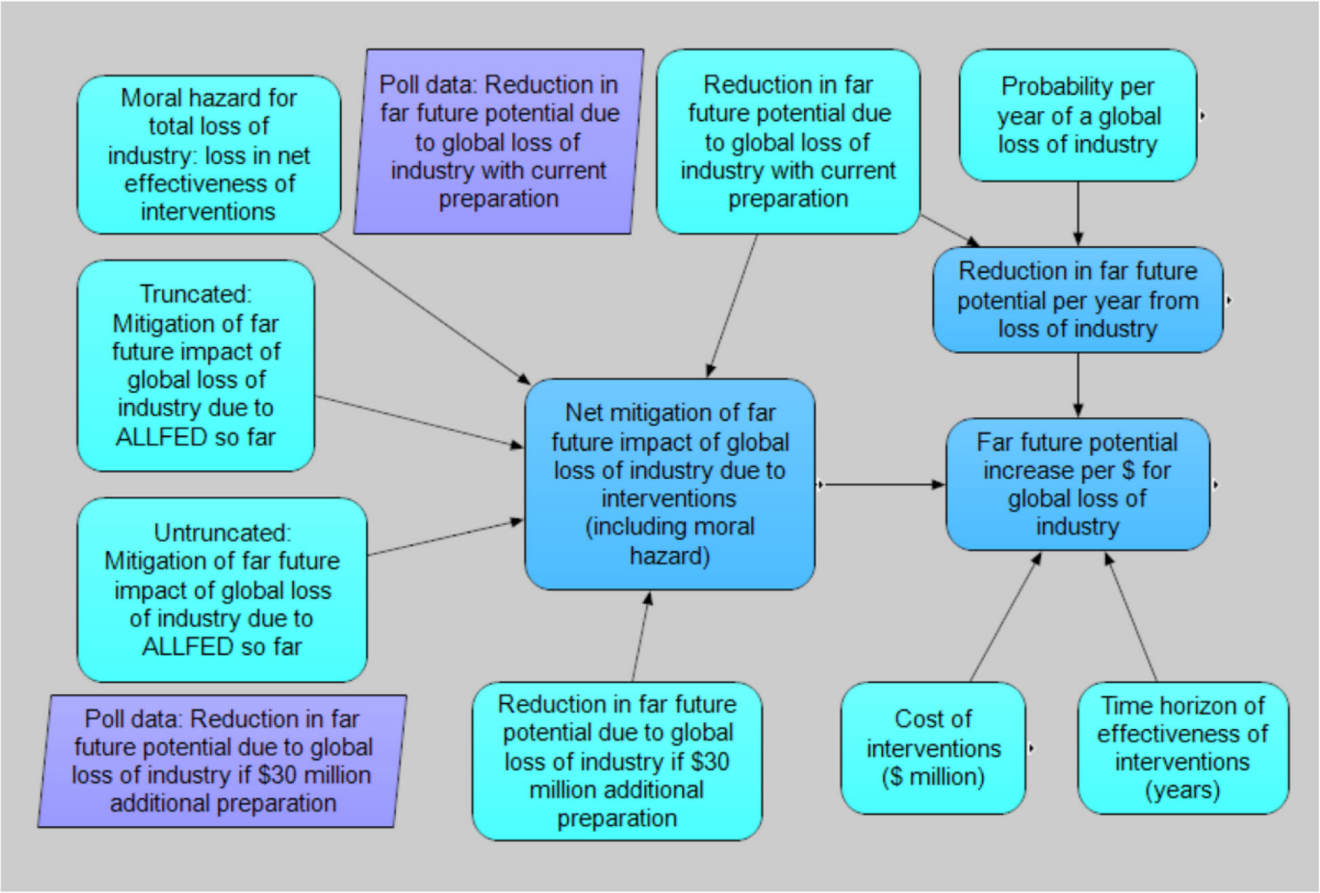
Global industry loss submodel (10% industry loss is nearly identical)

**Fig. 4:**
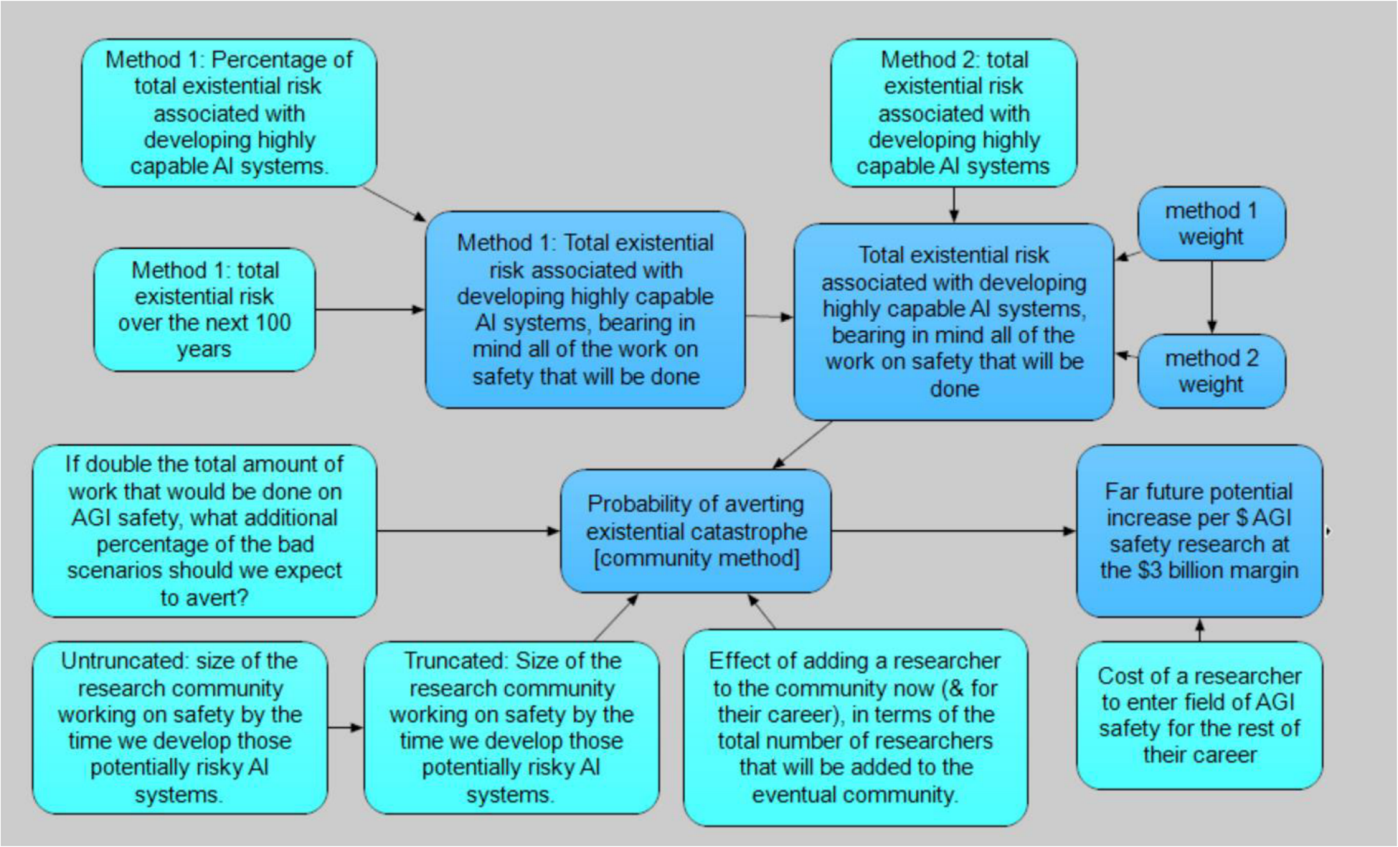
AGI safety cost-effectiveness submodel

**Fig. 5:**
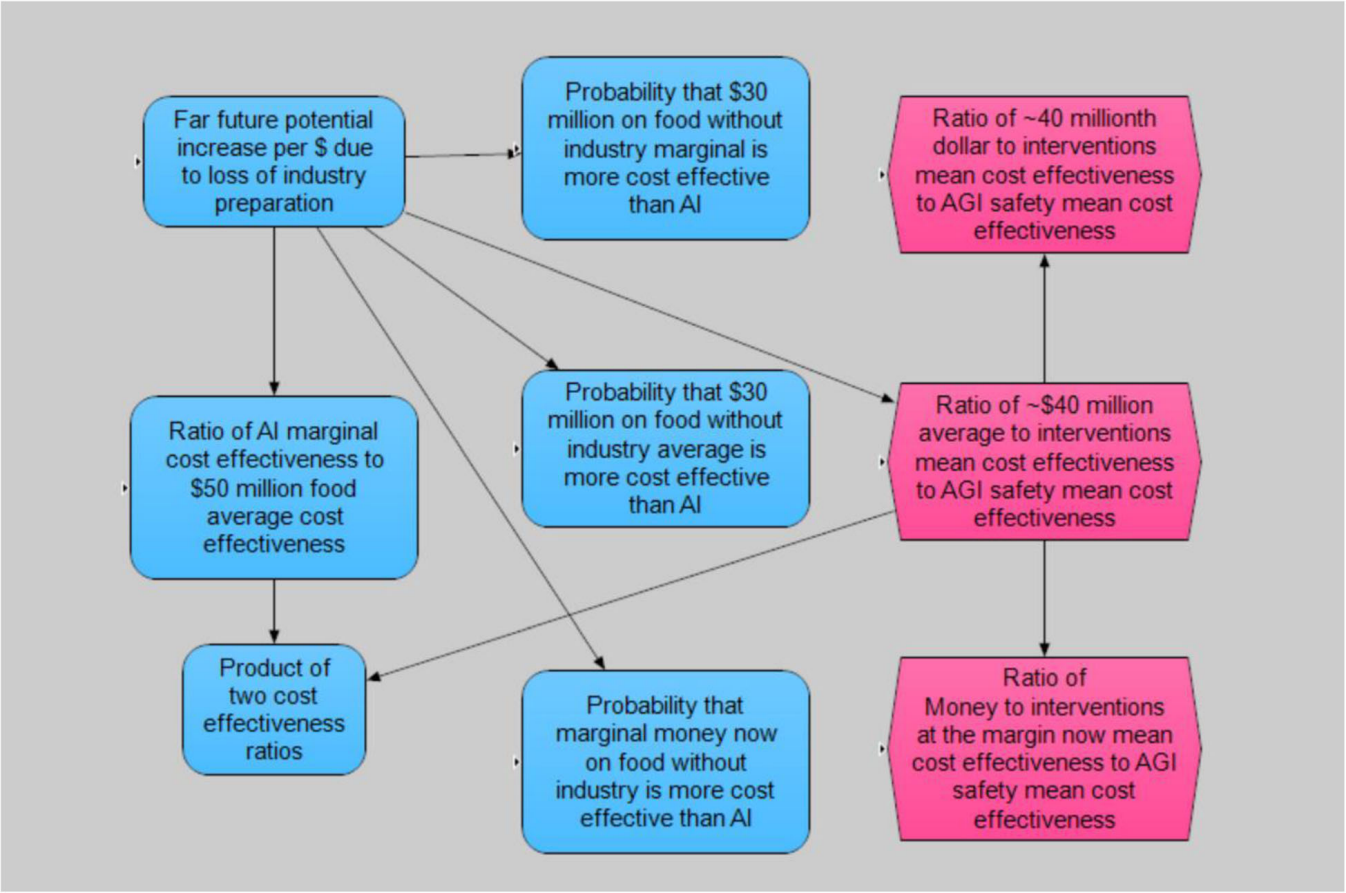
Overall cost-effectiveness ratios

Figures 2, 3, 4, and 5 illustrate the interrelationships of the nodes for model 1; model 2 is identical with the following exception. The input variable mitigation of far future impact of industrial loss from ALLFED so far for 10% industrial loss node was removed from model 1 due to the poll question not requiring this input.

### Loss of industry interventions submodel

Table 1 shows the key input parameters for model 1 (largely Denkenberger and conference poll of effective altruists) [40] and model 2 [38]) (Sandberg inputs).^3^ Though the authors here are associated with research on loss of industry, two out of four also published in AGI safety. Also, opinions outside of the loss of industry field have been solicited for one of the models. Therefore, we believe the results are representative. All distributions are lognormal unless otherwise indicated. The absolute value of the long term future is very difficult to quantify, so losses are expressed as a percent.

**Table 1:** Losing industry interventions input variables

Input variable	Model 1		Model 2	
	5th percentile	95th percentile	5th percentile	95th percentile
Cost of interventions ($ million)	10	70	5	150
Time horizon of effectiveness of interventions (years)	10	50	5	50
Probability per year of a global loss of industry	0.01%	1%	0.05%	0.15%
Probability per year of a 10% loss of industry	0.1%	10%	0.1%	1%
Reduction in far future potential due to global loss of industry with current preparation	3%	30%^a^	5%	10%
Reduction in far future potential due to 10% loss of industry with current preparation	0.03%	40%	0.001%	0.1%
Mitigation of far future impact of global loss of industry due to ALLFED so far	0.003%	0.3%	0.001%	0.01%
Mitigation of far future impact of 10% industrial loss from ALLFED so far	–	–	0.001%	0.01%
Reduction in far future potential due to global loss of industry if $30 million additional preparation	0.3%	30%		
Mitigation of far future impact of 100% industrial loss with planning and R&D as well			1%	5%
Mitigation of far future impact of 10% industrial loss with planning and R&D as well	3%	30%	2%	10%
Moral hazard for total loss of industry: loss in net effectiveness of interventions	− 3%	3%	0%	0%
Moral hazard for 10% industrial loss: loss in net effectiveness of interventions	− 3%	3%	0%	0%

^a^The global loss poll gave people ranges, including < 0.1%, 0.1% to 1%, 1% to 10%, and 10% to 100%. All responses in the range were recorded as approximately the geometric mean of the range. Half of people were therefore recorded as 30% loss of the far future. If the people had been able to provide exact values, likely one of them would have recorded greater than 40%, which was the upper bound for the 10% loss of industry, making these results consistent. However, even with the constraints of the data, the mean and median are higher for the global loss of industry than the 10% loss of industry

The potential causes of the disabling of 1/10 of global industry include Carrington-type solar storm, single HEMP, coordinated physical or cyber attack, conventional world war, loss of the Internet, and pandemic disrupting trade. We are not aware of quantitative estimates of the probability of a coordinated cyber attack, loss of the Internet, a pandemic that significantly disrupts trade, or a conventional world war that destroys significant industry and does not escalate to the use of nuclear weapons. Quantitative model estimates of the probability of full-scale nuclear war between the USA and Russia such as [13]) may give some indication of the probability of HEMP. HEMP could accompany nuclear weapons destroying cities, and this would be a combination losing industry/losing the sun scenario, which would benefit from the preparation considered here. Asymmetric warfare, where one country is significantly less powerful than another, could use HEMP because it only requires one or two nuclear weapons to disable an entire country. There are significantly more nuclear pairs that could result in HEMP than could result in full-scale nuclear war (the latter is basically the dyads between USA, Russia, and China). And yet one quantitative model estimate of the probability of full-scale nuclear war only between the USA and Russia was 1.7% per year (mean) [13]. In 2012, there was a near miss of a solar storm similar size to the Carrington event [11]. One probability estimate of a Carrington-sized event is ~ 0.033% per year [109]. However, an estimate of the probability per year of a superflare 20 times as powerful as the Carrington event is 0.1%/year [79], which disagrees by orders of magnitude for the same intensity. Another study proposes that a Carrington-sized event recurrence interval is less than one century [62]. Given the large uncertainty of solar storms and significant probability of single EMP, pandemic, and regional cyber attack, model 1 uses a mean of 3% per year. Model 2 uses a mean of 0.4% per year.

Intuitively, one would expect that the probability of near-total loss of industry would be significantly lower than 10% loss of industry. Complete loss of industry may correspond to the superflares that may have occurred in the first millennium A.D. (~ 0.1% per year). We are not aware of quantitative estimates of the probability of multiple EMP, industry-halting pandemic, or global cyber attack. Model 1 mean is 0.3% per year for near-total loss of industry. Model 2 mean is 0.09% per year.

At the Effective Altruism Global 2018 San Francisco conference, with significant representation of people with knowledge of existential risk, a presentation was given and the audience was asked about the 100% loss of industry catastrophes. The questions involved the reduction in far future potential due to the catastrophes with current preparation and if ~ $30 million were spent to get prepared. The poll questions and results are available in Table 6 (Appendix 3). The data from the poll was used directly instead of constructing continuous distributions for input nodes. The n responses per probability range were converted to point values equal to the geometric mean (rounded to one significant figure) of the probability range to create a list of values for each input node, which was randomly drawn from each run.

To determine the marginal impact of additional funding, the contribution due to work so far should be quantified. The Alliance to Feed the Earth in Disasters (ALLFED) [4] (and ALLFED researchers before the organization was officially formed) have published several papers on interventions for losing industry. They have a website with these papers and summaries. They have also run workshops to investigate planning for these interventions. However, we expect the contribution of ALLFED to reducing the long term impact of loss of industry to be significantly lower than in the case of obscuring of the sun because the loss of the Internet may be immediate if there are multiple simultaneous EMPs. However, the loss of electricity may not be simultaneous globally due to cyber-attack. Furthermore, there may be several days warning for an extreme solar storm. The other reason why current work may be less valuable in a global loss of industry scenario is that fewer people know about the loss of industry work of ALLFED than the food without the sun work. Model 1 estimates a reduction in long-term future potential loss from a global loss of industry due to ALLFED so far as a mean of 0.1%. Model 2 uses 0.004% due to emphasizing lack of communication scenarios.

In the case of a 10% loss of industry, with the exception of the scenario of loss of Internet everywhere, the Internet in most places would be functioning. Even if the Internet is not functioning, mass media would generally be functioning. Therefore, possible mechanisms for impact due to work so far include the people already aware of the interventions getting the message to decision makers/media in a catastrophe, decision makers finding the three papers [1, 39, 28] on these interventions, or the people in the media who know about these interventions spreading the message. However, even though people outside of the affected countries could get the information, it may not be feasible to get the information to the people who need it most. Model 2 estimates a reduction in long-term future potential loss from a global loss of industry due to ALLFED so far as a mean of 0.004%, again due to the likely lack of communications in the affected region. Model 1 does not use a value in its calculation.

The mean estimate of the conference participants was 16% reduction in the long-term future of humanity due to loss of global industry with current preparedness. Model 2 estimate mean was 7%.

The 10% industry loss catastrophes could result in instability and full scale nuclear war or other routes to far future impact. Though the poll was not taken for this level of catastrophe, a survey of GCR researchers estimated a mean of 13% reduction in long-term potential of humanity due to a 10% food shortfall [42]. Confidence in estimates solicited in the survey were not incorporated into the model (relevant survey question and responses are attached as Appendix 4). Some 10% loss of industry catastrophes could cause a ~ 10% global food shortfall. However, if the affected area were largely developed countries, since they would likely need to become near vegan to survive, human-edible food demand could fall 10% because of the reduction of feeding animals. Still, given the possible overlap of these catastrophes, this analysis uses the survey estimate for model 1. Model 2 estimate mean is 0.4% reduction in long-term potential due to 10% loss of industry.

The means of the percent further reduction in far future loss due to global loss of industry due to spending ~ $30 million were 40% for the poll and 3% for model 2. Note that in model 1, the poll did not ask for the further reduction in far future loss from spending money, but instead a new far future loss after the money was spent. Therefore, the 40% mean further reduction is a calculated value and does not appear in Table 1. For the 10% industrial shortfalls, our estimate of the mean reduction is 12% for model 1 because the contribution of additional spending on the aid from outside the affected region would be smaller. On the other hand, it was 5% for model 2 because he thought the likelihood of success would be greater than for the global loss of industry given the outside aid.

Moral hazard would occur if awareness of interventions makes catastrophes more likely or more intense. Global use of EMP or coordinated cyber attack could be perpetrated by a terrorist organization trying to destroy civilization. However, if the organization knew of backup plans that could maintain civilization, the terrorist might actually be deterred from attempting such an attack. This would result in negative moral hazard (additional benefit of preparation). However, it is possible that knowledge of a backup plan could result in people expending less effort to harden systems to EMP, solar storm, or cyber attack, creating moral hazard. Therefore, model 1 uses a mean moral hazard of zero, and model 2 uses a point value of zero.

For the 10% loss of industry scenarios, the same moral hazard values are used as for the global loss of industry.

### Costs of interventions

The costs of the proposed interventions are made up of a backup communication system, developing instructions, and testing them for distributed food production, and making response plans at different levels of governments.

Currently, the long distance shortwave radio frequencies are used by government and military stations, ships at sea, and by amateur (ham) radio operators. Because of security considerations, data on the number of government/military stations is difficult to compile. The use by ships has declined because of the availability of low cost satellite phones but there are an estimated three million ham operators worldwide [115]. Not all of those are licensed to use the shortwave bands, however. In the USA, about half of the approximately 800,000 American ham operators do hold the necessary license. Assuming such a pattern worldwide that would mean potentially about 1.5 million ham radio shortwave stations globally.

However, this analysis conservatively ignores the possibility that there would be existing ham radios that are disconnected with unplugged backup power systems. Therefore, the cost of the backup communication system of 5 million USD is based on the cost of 10 larger two-way shortwave communication systems (with backup power) that can transmit across oceans (see Appendix 2). Then there would be 4000 smaller one-way shortwave receivers (with backup power) that, when connected to a laptop computer and printer, would have the ability to print out information. This could be called REtaining Civilization Using Radio (RECUR). This would cover 80% of the world’s population within 1 day nonmotorized transportation distance (~ 40 km) according to geographical information systems (GIS) analysis (Fist et al., unpublished results). It is critical to very quickly get the message out that there is a plan and not to panic. Subsequent communication would be instructions for meeting basic needs immediately like food, shelter, and water. This initial planning would be considered open-loop control because it would not have immediate feedback [80].

In the ensuing months, as reality always deviates from plans, feedback would be required. This could be accomplished by coordinating additional undamaged shortwave and electrical generation equipment to allow two-way communication for many cities. Also, depending on distance, some messages could be communicated through non-electronic means such as horses, smoke signals, and sun reflecting heliographs of the kind that were used in the Western USA before telegraphs [108, 119].

Instructions would include how to get safe water or treat it (e.g., by filling containers including cleaned bathtubs with water in water towers and treating with bleach for a limited amount of time, solar water pasteurization [22], or boiling. Additional instructions would be on how to keep warm if it is cold outside [1]. Other instructions would be how to retrofit a light duty vehicle to be pulled by a large animal. Because cattle and horses can eat food that is not edible to humans and because the wheel is so efficient, this would be a much more effective way of moving people than people walking. Additional instructions would be how to create wood-burning stoves and hand and animal farming tools, e.g., from repurposed or landfill materials. A similar project is Open Source Ecology, where blueprints have been developed of essential equipment for civilization that can be made from scratch [98]. All of this should be tested on realistically untrained people and the instructions should be modified accordingly.

Planning involves determining where different people would need to be relocated in order to have their basic needs met. The critical short-term factors are shelter and water, while food is slightly longer term. The economically optimal plan could be achieved with GIS analysis. However, in order for this to be politically feasible, there would need to be negotiations and precommitments. This may have similar cost to the government planning for food without the sun of $1 million to $30 million [34].

Overall, model 1 estimates the communications, instructions/testing, and planning for global industry loss would cost roughly 30 million USD (see Table 1). For the regional loss of industry, it is difficult to predict where it might occur, so generally communications and planning should be done for the entire world, and thus the instructions/experiments would be similar. Therefore, there is a high correlation of preparation for the two catastrophes, so this is assumed to be the cost of the preparation to both scales of catastrophe. Model 2 has somewhat higher costs ($50 million mean).

The time horizon of effectiveness of the interventions would depend on the intervention. Modern shortwave radio communications equipment has few moving parts (chiefly cooling fans and motors to rotate directional antennas) and serviceability measured in decades.^4^

Furthermore, these systems need to be disconnected from the grid to be protected from HEMP. This would reduce wear and tear, but regular testing would be prudent. Some of the budget could be used for this and for repair of the units. As for the instructions, since the hand and animal tools are not changing, directions should stay relevant. Planning within governments is susceptible to turnover, but some money could be used to transfer the knowledge to new employees. Model 1 estimates a 25 year mean for the time horizon. Model 2 has a slightly shorter time horizon mean of 20 years driven by a conservative estimate of the communications equipment lifetime.

### Artificial intelligence submodel

The submodel for AGI safety cost-effectiveness was based on work of the Oxford Prioritization Project, Owen Cotton-Barratt and Daniel Dewey (both while at the Future of Humanity Institute at the University of Oxford) [41, 78]. We modified it with major changes including increasing the cost of an AGI safety researcher, making better behaved distributions, removing one method of calculation, and changing the analysis from average to marginal for the number of researchers. These changes increased the cost-effectiveness of AGI safety by roughly a factor of two and increased the uncertainty considerably (because the method of calculation retained had much greater uncertainty than the one removed). The cost-effectiveness was found at the margin assuming $3 billion expenditure.

## Results and discussion

### Results

In order to convert average cost-effectiveness to marginal for interventions, we use logarithmic returns [30], which results in the relative marginal cost-effectiveness being one divided by the cumulative money spent. An estimate is needed of the cumulative money spent so far for interventions. Under $100,000 equivalent (mostly volunteer time) has been spent so far directly on this effort, nearly all by ALLFED. A very large amount of money has been spent on trying to prevent nuclear war, hardening military installations to HEMP, and on cyber security. However, note that even though US military infrastructure is supposedly hardened to EMP, it may not be able to withstand a “super” EMP weapon that some countries may possess [105] or sophisticated cyber attacks. More relevant, money has been spent on farming organically and less industrially for traditional sustainability reasons. Also, Open Source Ecology has developed instructions for critical equipment. These could be tens of millions of dollars that would have needed to be spent for catastrophe preparation. So this would be relevant for the marginal $30 million case. However, there are still very high value interventions that should be done first, such as collecting instructions for producing hand/animal farm tools without industry and giving them to at least some governments and owners of disconnected shortwave radios and backup power sources. Though the interventions would not work as well as with ~ $30 million of research/communications backup, simply having some critical people know about them and implement them in their own communities/countries without trade could still significantly increase the chance of retaining anthropological civilization. The cost of these first interventions would be very low, so they would have very high cost-effectiveness.

**Fig. 6:**
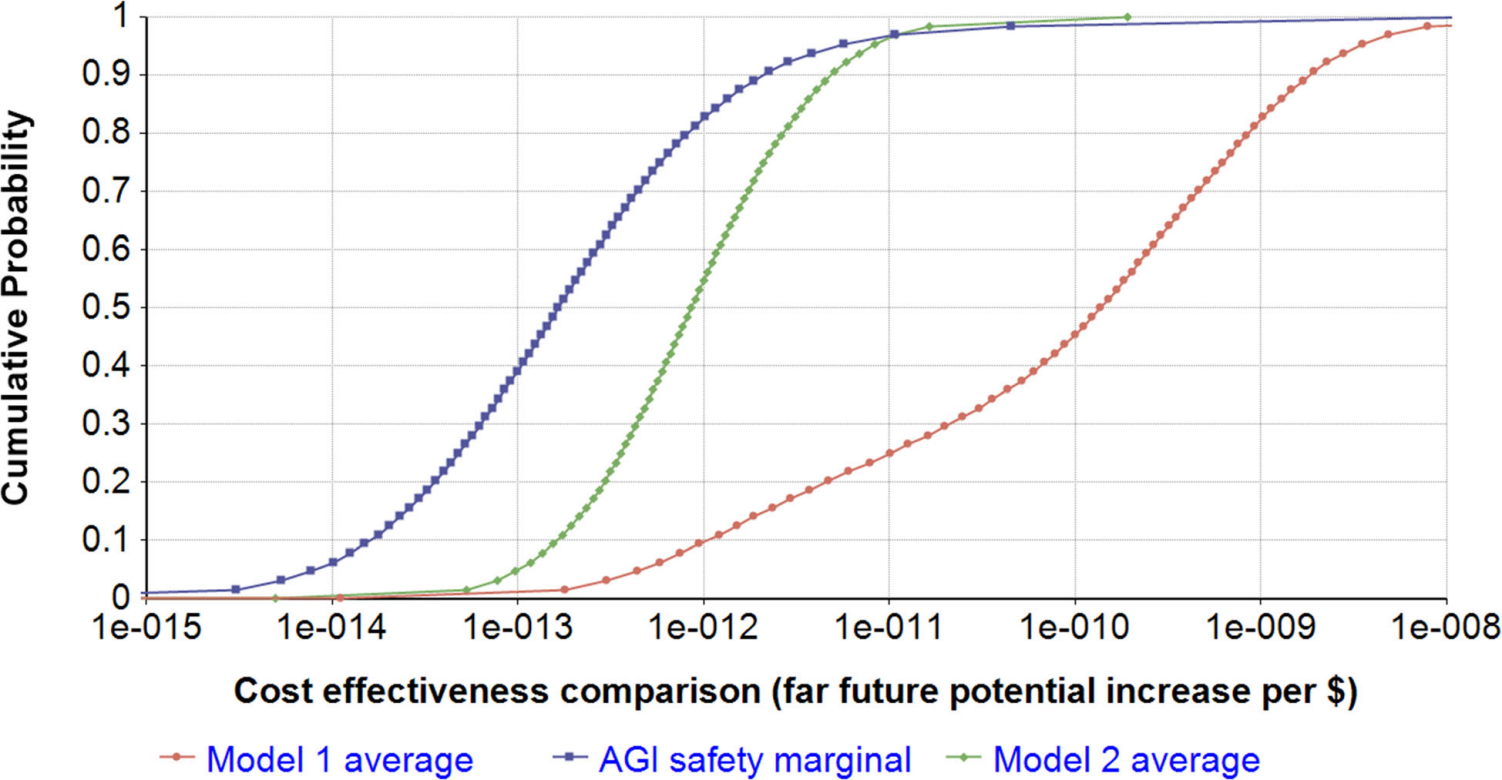
Far future potential increase per $ due to loss of industry preparation average over ~ $30 million model 1, due to loss of industry preparation average over ~ $50 million model 2, and AGI safety research at the $3 billion margin. Further to the right is more cost-effective

Table 2 shows the ranges of the far future potential increase per $ due to loss of industry preparation average over ~ $30 for million model 1, average over ~ $50 million for model 2, and AGI safety research at the $3 billion margin. The distributions are shown in Fig. 6. The ratios of the 95th and 5th percentiles of model 1, model 2, and AGI safety are 8000, 80, and 600, respectively. Because the variance of model 1 is very high, the mean cost-effectiveness is high, partly driven by the small probability of very high cost-effectiveness.

**Table 2:** Cost-effectiveness comparison

Output	5th percentile	95th percentile
Far future potential increase per $ due to loss of industry preparation average over ~ $30 million model 1	4E-13	3E-9
Far future potential increase per $ due to loss of industry preparation average over ~ $50 million model 2	1E-13	8E-12
Far future potential increase per $ AGI safety research at the $3 billion margin (same for both models)	8E-15	5E-12

With logarithmic returns, cost-effectiveness of the marginal dollar now (100,000th dollar) and of the last dollar are about 50 times greater than, and 6 times less than, the average cost-effectiveness of spending $30 million, respectively. For model 2, the corresponding numbers are about 70 times greater than and 6 times less than the average cost-effectiveness of spending $50 million. Ratios of mean of the distributions of cost-effectiveness are reported in Table 3.^5^ Comparing to AGI safety at the margin, model 1 yields the 30 millionth dollar on losing industry being 20 times more cost-effective, the average $30 million on interventions being 100 times more cost-effective, and the marginal dollar now on interventions being 5000 times more cost-effective (Table 3). Model 2 yields the last dollar on interventions being 0.05 times as cost-effective, the average ~ $50 million on interventions being 0.2 times as cost-effective, and the marginal dollar now on interventions being 20 times as cost-effective. Given orders of magnitude uncertainty and sensitivity of these ratios to the relative uncertainty of the interventions, likely more robust are the probabilities that one is more cost-effective than the other. Comparing to AGI safety at the margin, model 1 finds ~ 88% probability that the 30 millionth dollar on interventions is more cost-effective, ~ 95% probability that the average $30 million on interventions is more cost-effective, and ~ 99+% probability that the marginal dollar now on interventions is more cost-effective (see Table 3). Model 2 finds ~ 50% probability that the 50 millionth dollar on interventions is more cost-effective than AGI safety, ~ 76% probability that the average $50 million on interventions is more cost-effective, and ~ 99% probability that the marginal dollar now on interventions is more cost-effective. Note that the greater than 50% probability for the average cost effectiveness despite the ratio of the means of cost-effectiveness being less than one is due to the relatively smaller variance of model 2 cost-effectiveness estimate (see Fig. 6).

**Table 3:** Key cost-effectiveness outputs of losing industry interventions

Scenario	Model 1		Model 2	
	Ratio of interventions mean cost-effectiveness to AGI safety mean cost effectiveness	Confidence that interventions are more cost-effective than AGI safety	Ratio of interventions mean cost-effectiveness to AGI safety mean cost effectiveness	Confidence that interventions are more cost-effective than AGI safety
~ 40 millionth dollar to interventions	20	88%	0.05	50%
~ $40 million average to interventions	100	95%	0.2	76%
Money to interventions at the margin now	5000	99+%	20	99%

Overall, the mean cost-effectiveness of model 1 is about 2.5 orders of magnitude higher than model 2. However, due to the smaller variance in model 2 distributions, there was similar confidence that losing industry interventions at the margin now are more cost-effective than AGI safety. Another large difference is that model 1 found that 10% loss of industry scenarios are similar cost-effectiveness for the far future as global loss. This was because the greater probability of these catastrophes counteracted the smaller far future impact. However, model 2 rated the cost-effectiveness of the 10% industry loss as ~ 1.5 orders of magnitude lower than for global loss. Given the agreement of high confidence that further work is justified at this point, some of this further work could be used to resolve the significant uncertainties to determine if more money is justified: value of information [12].

Being prepared for loss of industry might protect against unknown risks, meaning the cost-effectiveness would increase.

According to model 1, every year acceleration in preparation for losing industry would increase the long-term value of humanity by 0.00009% to 0.4% (mean of 0.07%). The corresponding model 2 numbers are 0.00006% to 0.0004% (mean of 0.00017%). Either way, there is great urgency to get prepared.

It is not necessary for interventions to be more cost-effective than AGI safety in order to fund losing industry interventions on a large scale. Funding in the existential risk community goes to other causes, e.g., an engineered pandemic. One estimate of cost effectiveness of biosecurity was much lower than for AGI safety and losing industry interventions, but the authors were being very conservative [86]. Another area of existential risk that has received investment is asteroid impact, which again has much lower cost-effectiveness than for losing industry interventions [83].

The importance, tractability, neglectedness (ITN) framework [45] is useful for prioritizing cause areas. The importance is the expected impact on the long-term future of the risk. Tractability measures the ease of making progress. Neglectedness quantifies how much effort is being directed toward reducing the risk. Unfortunately, this framework cannot be applied to interventions straightforwardly. This is because addressing a risk could have many potential interventions. Nevertheless, some semi-quantitative insights can be gleaned. The importance of AGI is larger than industry loss catastrophes, but industry loss interventions are far more neglected.

Though these interventions for the loss of industry are not compared directly to food without the sun interventions, they are both compared to the same AGI safety submodel. Overall, model 2 indicates that spending $50 million on interventions for the loss of industry is competitive with AGI safety. However, model 1 here and both models for the food without sun indicate that significantly larger than the proposed amount to be spent (~ $100 million) would be justified from the long-term future perspective.

The AGI safety submodel was used to estimate the cost-effectiveness of saving expected lives in the present generation, finding $16–$12,000 per expected life saved (Author et al., unpublished results). This is generally more cost-effective than GiveWell estimates for global health interventions: $900–$7000 [52]. Food without the sun is significantly better ($0.20–$400 per expected life) for only 10% global food production shortfalls [34] and generally better only considering one country ($1–$20,000 per expected life) and only nuclear winter [34]. Model 2 for interventions for losing industry has similar long-term future cost-effectiveness to AGI safety, indicating that the lifesaving cost-effectiveness of interventions for losing industry would likely be competitive with AGI safety and global health, but this requires future work. Model 1 for interventions for losing industry has similar long-term future cost-effectiveness to food without the sun, indicating that loss of industry preparations may save lives in the present generation less expensively than AGI safety and global health. Since AGI safety appears to be underfunded from the present generation perspective, it would be extremely underfunded when taking into account future generations. If this were corrected, then in order for interventions for losing industry to stay similar cost-effectiveness to AGI safety, more funding for losing industry interventions would be justified.

### Timing of funding

If one agrees that interventions for losing industry should be a significant part of the existential risk reduction portfolio, there remains the question of how to allocate funding to the different causes over time. For AGI safety, there are arguments both for funding later and funding now [99]. For interventions for losing industry, since most of the catastrophes could happen right away, there is significantly greater urgency to fund interventions for losing industry now. Furthermore, it is relatively more effective to scale up the funding quickly because, through requests for proposals, the effort could co-opt relevant existing expertise (e.g., in shortwave radio). Since we have not monetized the value of the far future, we cannot use conventional cost-effectiveness metrics such as the benefit to cost ratio, net present value, payback time, and return on investment. However, in the case of saving expected lives in the present generation for the global case and 10% food shortfalls, the return on investment was from 100 to 5,000,000% per year [34] based on monetized life savings. This suggests that the $40 million or so for interventions for losing industry should be mostly spent in the next few years to optimally reduce existential risk (a smaller amount would maintain preparedness into the future).

### Uncertainty and parameter sensitivity

Parameter sensitivities of model 1 and model 2 were investigated using the Analytica importance analysis function. This uses the absolute rank-order correlation between each input and the output as a measure of the strength of monotonic relations between each uncertain input and a selected output, both linear and otherwise [25, 87]. Analysis was focused on the loss of industry submodels, i.e., global loss of industry and 10% industry loss. Parameter sensitivity within AGI safety was not investigated as this submodel was adapted from previous work by the Oxford Prioritization Project, which discussed uncertainties within the AGI safety cost effectiveness submodel ([41, 78]).

The key outputs nodes in Table 3 were unable to be investigated directly using the importance analysis function due to the node outputs being point values, a result of calculating the confidence or ratio of means (the Analytica importance analysis function requires the variable be a chance variable to perform absolute rank-order correlation). Therefore, the previous node in the models’ far future potential increase per $ overall was used to investigate the importance of input variables of the alternate foods submodel.

**Fig. 7:**
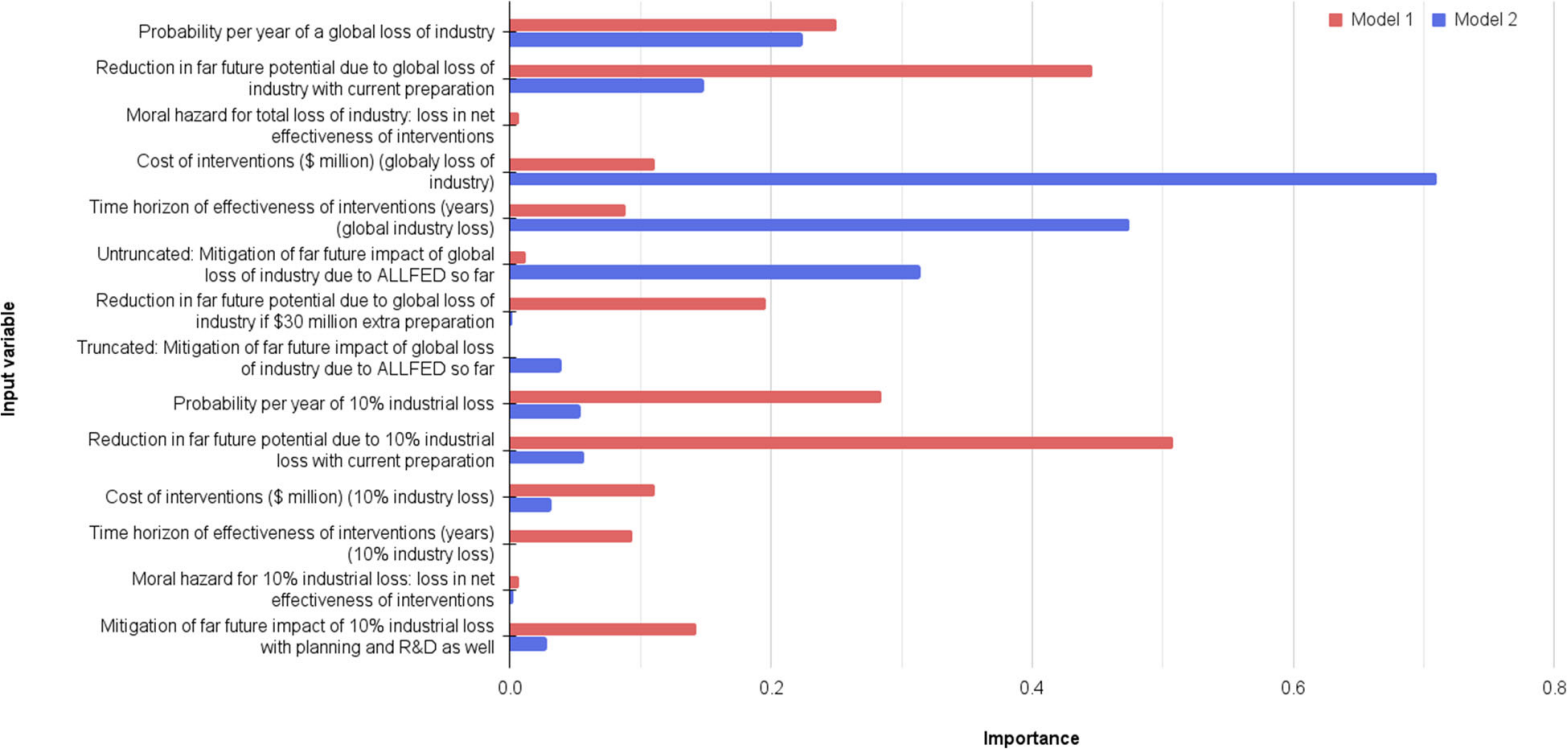
Importance analysis results for Far future potential increase per $ due to loss of industry preparation for model 1 and model 2

Importance analysis of node: far future potential increase per $ due to loss of industry preparation showed model 1 had greatest sensitivity to input variables reduction in far future potential due to 10% industrial loss with current preparation closely followed by reduction in far future potential due to global loss of industry with current preparation (Fig. 7). Model 2 showed greatest sensitivity to input variable cost of interventions ($ million) (global loss of industry) (Fig. 7).

Successive rounds of parametric analysis were performed to determine combinations of input parameters sufficiently unfavorable to losing industry interventions, until cost-effectiveness ratios (Table 3) switched to favoring AGI safety. Unfavorable input values were limited to 5th or 95th percentile values of original input distributions. Model 1 required 7 unfavorable input parameters to switch to AGI safety being more cost-effective than losing industry interventions at the margin now while model 2 required 4 input variables (see Table 4).

**Table 4:** Combination of input variables resulting in AGI safety being more cost effective than losing industry interventions at the margin now

Input variable	Model 1	Model 2
Cost of interventions ($ million) global industry loss	70	5
Time horizon of effectiveness of interventions (years) global industry loss	10	5
Probability per year of a global loss of industry	1E-4	5E-4
Probability per year of a 10% loss of industry	0.001	–
Reduction in far future potential due to global loss of industry with current preparation	0.03	–
Reduction in far future potential due to 10% loss of industry with current preparation	3E-4	–
Mitigation of far future impact of 100% industrial loss with planning and R&D as well	–	0.01
Mitigation of far future impact of 10% industrial loss with planning and R&D as well	0.03	-
Output variable	Model 1	Model 2
Ratio of money to interventions at the margin now mean cost effectiveness to AGI safety mean cost effectiveness	0.72	0.64

A robustness analysis was not performed for the confidence values of one type of intervention being likely to be more cost-effective than the other. This would be less sensitive to the variance in the distributions than the ratios of the mean cost-effectiveness. Since the variance in model 1 is so large, it would require fewer variables to be made pessimistic in order for it to be less than 50% confident that it is more cost-effective than AGI safety (than the number of variables required for the ratio of the means in Table 4). Conversely, since the variance in model 2 is smaller than for AGI safety, it would require fewer variables to be made pessimistic in order for it to be less than 50% confident that it is more cost-effective than AGI safety.

## Conclusions and future work

There are a number of existential risks that have the potential to reduce the long-term potential of humanity. These include AGI and electricity/industry disrupting catastrophes including extreme solar storm, EMP, and coordinated cyber attack. Here, we present the first long-term future cost-effectiveness analyses for interventions for losing industry. There is great uncertainty in both AGI safety and interventions for losing industry. However, the models have 99–99+% confidence that funding interventions for losing industry now is more cost effective than additional funding for AGI safety beyond the expected $3 billion. In order to make AGI safety more cost effective than losing industry interventions according to the mean of their distributions, this required changing four variables in model 2 to the 5th percentile on the pessimistic end simultaneously. For model 1, it required changing seven variables. Therefore, it is quite robust that a significant amount of money should be invested in losing industry interventions now. There is closer to 50–88% confidence that spending the ~ $40 million on interventions for losing industry is more cost effective than AGI safety. These interventions address catastrophes that have significant likelihood of occurring in the next decade, so funding is particularly urgent. Both AGI safety and interventions for losing industry save expected lives in the present generation more cheaply than global poverty interventions, so funding should increase for both. The cost-effectiveness at the margin of interventions for the loss of industry is similar to that for food without the sun (for industry versus sun, model 1 is ~ 1 order of magnitude more cost effective, but model 2 is ~ 1 order of magnitude less cost effective). Because the electricity/industry catastrophes could happen immediately and because existing expertise relevant to food without industry could be co-opted by charitable giving, it is likely optimal to spend most of this money in the next few years.

Since there may be scenarios of people eating primarily one food, micronutrient sufficiency should be checked, though it would be less of an issue than for food without the sun [35, 59]. Higher priority future research includes ascertaining the number and distribution of unplugged shortwave radio systems with unplugged power systems that could be utilized in a catastrophe. Additional research includes the feasibility of the continuation of improved crop varieties despite loss of industry. Further research is estimating the rapidity of scale up of hand and animal powered farm tools. Estimating the efficacy of pest control without industry would be valuable. Better quantifying the capability of using fertilizer based on ash would be aided by GIS analysis. Additional work is surveying whether there have been experiments of the agricultural productivity produced by people inexperienced in farming by hand.

Another piece of future work would be to analyze the cost-effectiveness of AGI safety and preparation for the loss of industry in terms of species saved. Rogue AGI could cause the extinction of nearly all life on earth. If there were mass starvation due to the loss of electricity/industry, humans would likely eat many species to extinction. Therefore, being able to meet human needs would save species. These cost effectiveness could be compared to the cost effectiveness of conventional methods of saving species. Finally, additional future work involves better quantifying the cost of preparedness to the loss of industry. Furthermore, research for the actual preparedness should be done, including estimating the amount of unplugged communications hardware and backup power, testing the backup communications system, experiments demonstrating the capability to quickly construct hand/animal farm tools and developing quick training to use them. Also investigating alternative food sources that do not require industry would be beneficial, such as seaweed.

## Data Availability

Guesstimate models can be accessed via links available in the reference section of the manuscript. Analytica models are available from the corresponding author on reasonable request.

## Appendix 1

### Civilization collapse from severe pandemic

#### Complex scenario description

A severe pandemic could be caused without human involvement (i.e., a natural risk) or be caused by humans, whether intentionally (i.e., a malicious risk) or unintentionally (i.e., an accident risk). For instance, a mutation of COVID-19 with increased virulence and mortality could emerge [60] and go undetected before infecting multiple people (i.e., natural risk). Alternatively, a doomsday cult could become obsessed with designing the “perfect” virus [110] and be successful in their endeavor (i.e., malicious risk) thanks to easier availability of gene modification technologies such as CRISPR-CAS9 [24]. Furthermore, a biotechnology lab working with highly infectious diseases could accidently leak a virus (i.e., accident risk) that was created for research purposes [71]. No matter the actual origin, multiple plausible scenarios for the emergence of a highly infectious and deadly pathogen seem to exist. Moreover, humanity’s failure in keeping COVID-19 contained and from becoming a global pandemic is a vivid illustration of the very real possibility of future pathogens also spreading across the globe.

#### Potential impact logic

Figure 1 illustrates a potential impact logic for the outlined scenario. Information communication technology (ICT), the electrical grid, and health services are three first-order dependencies which rely on the human population being impacted as a result of the virus making critical staff unavailable. In particular, reliable functioning of those Critical Infrastructure CI requires a core of highly skilled personnel to undertake various operational tasks. During a global pandemic, this core of critical personnel could be rendered partially or near completely unavailable through death, illness, family care requirements, government public health recommendations, such as lock downs, fear, and/or quarantining due to suspected illness [82]. Depending on the severity of infectious disease (the combination of transmissibility and virulence), failure could be rapid or more gradual. A highly virulent infectious disease (high transmissibility, high lethality) could rapidly decrease staffing below required operating numbers resulting in a rapid system-wide shut down. A less infectious disease or one with a longer incubation time could result in a more gradual failure as chronic understaffing resulted in reduced maintenance and recovery capacity. Thus, these dependencies are loose, as failures result over weeks as more of the human population (and thus critical staff) become infected. This is as opposed to tight dependencies, such as blackouts, which manifest rapidly and have direct impacts.

Importantly, our analysis suggests that multiple feedback loops exist, such as between the human population, ICT, and the electrical grid. This feedback loop would result from critical staff shortages leading to grid failures, causing ICT to fail, which reduces availability of public health information, which in turn causes greater infection levels that further decrease staff availability for CI. Similarly, health services are loosely dependent on the electrical grid once backup generators run out of fuel, which could precipitate a feedback loop of more infections being caused due to reduced capacity of health services, which in turn causes more staff shortages in CI.

The ability of the infrastructure ecosystem to absorb such stresses introduced by a pandemic depends on the slack (looseness) in key dependencies. For instance, key parameters include how long the electrical grid, ICT infrastructure, and natural gas infrastructure could be chronically understaffed before a critical failure occurs. Once such failures occur, a cascade of failures in dependent systems could be initiated, resulting in critical transitions to system failures. A series of critical transitions across infrastructure systems may occur as the infrastructure ecosystem moves through various states of degradation, before coming to rest at a stable state. Intermediate stable states may exist due to the buffering capacity of a series of loose dependencies. In sum, the diagram highlights how a pandemic could facilitate Catastrophic electricity loss (CEL) by causing an extensive cascade through various interdependent CI. Note that only three first-order effects are shown to highlight a plausible mechanism by which a pandemic could cause CEL. In reality CI are highly interlinked and multiple causal chains exist.

For instance, global supply chain interruptions expected during pandemics would increase already long lead times for critical grid components such as extra high voltage (EHV) transformers and nuclear power station steam turbines, which are manufactured by a limited number of highly specialized companies [94, 133]. Production of EHV transformers requires niche input materials such as electrical steel that are made by a limited number of specialized steel manufacturers, which makes sourcing of alternative inputs during supply chain disruption extremely difficult [94]. Anticipated reductions in global trade during a pandemic^6^ are also expected to decrease availability of more general grid components, further hampering grid maintenance and repair efforts.

Refueling of nuclear power plants illustrates how supply chain disruptions and induced staff shortages could lead to difficult challenges. Refueling is undertaken by a small number of external vendors, requiring highly specialized staff to undertake hundreds of maintenance steps under stringent safety protocols for successful refueling. This process takes ~ 4 weeks and must be performed every 18–24 months depending on reactor design [131]. Nuclear power contributed approximately 841 billion kilowatt hours (TWh) or ~ 19% of US electrical consumption in 2018, across 94 reactors [134]. With such a significant amount of energy concentrated over a relatively small number of production facilities (8.9 TWh per year for the average reactor in 2018) delays beyond the anticipated ~ 4 week refueling would compound to produce significant reductions in energy generation. In order to prevent delays in refueling, healthy personnel would likely be required to work overtime increasing the potential for human error. Similar challenges apply to various other forms of power generation such as gas, coal, hydro-electric, etc.

Moreover, a severe pandemic could cause failures of portions of the electrical grid by inducing failure of critical interlinked systems, e.g., failure of fossil fuel production and distribution. For instance, in order for natural gas power plants to provide the 1558 TWh (37% of electrical energy consumed in the U.S. in 2018) they currently produce, the timely delivery of natural gas to power production infrastructure is required. Achieving this requires the correct functioning of a complex network of gas extraction, refining and distribution infrastructure, which too must be maintained by highly specialized personnel and equipment. For the same reasons as described above, i.e., the requirement for adequate staffing of skilled workers and functioning of supply chains for acquisition of specialized equipment, critical input systems for the electrical grid, such as fossil fuel, are vulnerable to pandemics.

An additional concern which merits increased vigilance for the risk of pandemics is the rise of international flights, drastically increasing rates of international travel between populations. Diseases with high mortality rates that would normally have petered out on their own may not do so with high rates of international travel. In a recent study involving a simple two-dimensional model of a high mortality infectious disease (such as Ebola), a sharp phase transition was found above a threshold level of international travel, where infection spreads to most hosts. The probability of extinction of the hosts increased to near certainty after the phase transition [14, 92]. Such a sharp transition may obscure how dangerously close a high mortality outbreak could be to causing extinction or near-extinction, especially if rates of international travel increase.

The severe health crisis of COVID-19 has demonstrated the willingness of countries to restrict international trade, with the potential to cause more deaths from food insecurity and starvation than the virus alone. Additionally, there have been significant stresses on logistics and manufacturing from the virus [53, 74, 116].

Though no major disruptions to electrical grids have resulted from the COVID-19 pandemic thus far, the potential for disruption has been discussed in the literature. Potential catalysts include disruption of critical interlinked systems such as global supply chains [121]; delays in obtaining inputs for energy production [46]; novel cyber security risks to communication and SCADA systems due to changing work protocols [46]; and staff shortages from illness and travel restrictions within countries and between countries impacting mutual assistance agreements after significant blackouts [44]. It is uncertain how COVID-19 will proceed over the coming months; however, as pandemics exhibit “fat tailed” statistical properties (i.e., there is a non-negligible chance of extreme outcomes) [26]; the potential for severe outcomes is plausible. Novel mutations of COVID-19 with increased transmissibility or mortality provide an illustrative example [92, 106]. Associated risks to electrical grids and other critical infrastructure systems would also increase from losses of skilled staff, and greater supply chain disruptions. The complex nature of interactions between the various critical infrastructure systems imply that non-linear relationships between an increasingly severe pandemic and effects on critical infrastructure should be expected [81].

### Plausibility of the scenario

The 2014–2016 Ebola outbreak was an example of an epidemic outbreak that could have turned into a catastrophic global pandemic, with dire effects on critical national infrastructures including agriculture and industry. The primary countries affected by the outbreak, Sierra Leone, Liberia, and Nigeria, had a mean case fatality rate of 50.7%, with a corrected reproductive rate of the virus of 1.64 (i.e., the average number of people a person currently infected with the virus will infect) in Sierra Leone and Liberia [68]. The result from this highly deadly outbreak was a drastic and rapid reduction in agricultural and mining output, with the two largest industries in Sierra Leone, Liberia and Nigeria experiencing major disruption. Within a year major agricultural industries were severely affected. Coffee production fell by half, cocoa production by one third, and palm oil production dropped 75% [2]. Moreover, early on in the epidemic, London Mining, Sierra Leone’s second largest iron ore producer, suspended its activities [5]. Despite these outcomes, Ebola only infected a few tens of thousands of people. A larger outbreak with a similarly deadly but more transmissible virus would likely have led to much more disruption.

### Potential interventions to reduce food system impacts

If there is an extended loss of electricity and the functioning of other CI, urgent needs of water and shelter need to be met first. A backup shortwave radio communication system would facilitate the timely advice on where to get water and possibly where to relocate to. Local food supply would likely run out within weeks, so movement of food to people or people to food would be required [1]. For a limited amount of time, this could use aboveground fossil fuels. But over a longer period of time, retrofitting vehicles to be pulled by animals would be useful. Also, adapting ships to be sail or kite powered would be valuable. Replacement of industrial fertilizers could be achieved by planting legumes (peas or beans that fix nitrogen from the air), and burning wood in landfills for other nutrients [28].

## Appendix 2

### Radio component costs—available on https://osf.io/rgq2z/

**Table:** **Table 5**

Radio hub equipment	S'rce	Qty	Price each	Total price
HF exciter ICOM IC-7300	1	10	$1,699.95	$16,999.50
Ameritron amplifier AL-80BXCE	2	10	$1,729.95	$17,299.50
Ameritron QSK-5x high speed relay	2	10	$419.95	$4,199.50
Ameritron ARB-704 xcver to amp interface	2	10	$59.95	$599.50
RS-BA1 v2 remote control software	1	10	$149.00	$1,490.00
Samlex SEC-1235M power supply	1	10	$173.30	$1,733.00
ICOM mini manual #2612	1	10	$24.95	$249.50
USB-RTS-01 usb to 3.5mm plug cable	1	10	$29.95	$299.50
ICOM SM-30 microphone	1	10	$195.95	$1,959.50
Pactor modem DR7800P4 Dragon	3	10	$1,999.00	$19,990.00
Pactor modem audio cable for Icom 7300	3	10	$39.00	$390.00
Pactor modem ctrl cable 9095 for Icom 7300	3	10	$49.00	$490.00
MFJ-989D 1.5 KW manual antenna tuner	1	10	$469.95	$4,699.50
MFJ-4230 power supply—30A	1	10	$99.95	$999.50
MFJ-259C antenna analyzer	2	10	$319.95	$3,199.50
MFJ—1704 antenna switch	2	10	$79.95	$799.50
MFJ-250 dummy load	2	10	$49.95	$499.50
MFJ-21 transformer oil	2	10	$29.95	$299.50
MFJ-272 lightning protector	2	10	$39.95	$399.50
MFJ-97H replace't gas disch tubes-set of 5	2	10	$99.75	$997.50
cables, wire, connectors and fittings – set	1	10	$700.00	$7,000.00
Cushcraft A4S 3-band rotatable antenna	4	10	$759.95	$7,599.50
Rotator Hygain HAMIV	1	10	$709.95	$7,099.50
Tower 50' Rohn 25G90R050	5	10	$3,492.99	$34,929.90
Hustler BTV6 vert antenna—x 2 phased	1	10	$270.00	$2,700.00
Grand total—hub transmitting equipment				$136,922.40
**Miscellaneous equipment**				
solar backup electric system	7	10	$8,000.00	$80,000.00
laptop computer	6	10	$500.00	$5,000.00
Set of handtools including power drill,saw,etc	6	2	$400.00	$800.00
UPS Ref. CyberPower CP1500 PFC type	6	10	$199.81	$1,998.10
Total miscellaneous hub equipment				$87,798.10
**Grand total—all hub equipment**				**$224,721**
**Shortwave receive only downlinks**				
**Receiver/printer**				
AM-FM-Shortwave receiver	7	4000.00	$250.00	$1,000,000.00
Converter box	7	4000.00	$100.00	$400,000.00
Laptop	6	4000.00	$150.00	$600,000.00
Printer	6	4000.00	$100.00	$400,000.00
Total- shortwave receiving and printing			$600.00	$2,400,000.00
PV electric power system	7	4000.00	$600.00	$2,400,000.00
Total—receiver downlinks w/backup power		4000.00	$1,200.00	$4,800,000.00
**Grand total—RECUR network**				$5,024,720.50
Equipment Sources				
1 Universal Radio, Worthington, OH				
www.universal-radio.com				
2 MFJ Enterprises, Starkville, MS				
www.mfjenterprises.com				
3 Farrallon Electronics, San Rafael, CA				
www.farrallon.us				
4 Cushcraft Antennas, Starkville, MS				
cushcraftamateur.com				
5 DX Engineering, Tallmadge, OH				
www.dxengineering.com				
6 Amazon				
amazon.com				
7 miscellaneous				
1. Solar electric systems assume intermittent use of 1kw transmitters				
2. Prices for Icom transceivers and Ameritron amplifiers are list and may be different at time of purchase				
3. Does not include taxes, shipping, insurance, import duties, c				

## Appendix 3

### Effective Altruism Conference poll questions

**Table 6:** Poll results from Effective Altruism Global 2018

Question	> 10%	1–10%	0.1–1%	< 0.1%
What is your estimate of the loss in far future potential due to a catastrophe that disables industrial civilization globally with current preparation?	8	8	0	0
What is your estimate of the loss in far future potential due to a catastrophe that disables industrial civilization globally with an additional $30 MM USD spent on preparation?	3	13	2	0

## Appendix 4

### Email survey of GCR researchers on loss of civilization

Below is the informational text from the survey:

Earth-derived civilization has potential to spread to the galaxy and possibly beyond. Global catastrophes could reduce the potential of this civilization directly or with further conflict through risk of extinction, risk of losing industrial civilization and not recovering, risk of losing anthropological civilization (basically cities) and not recovering both anthropological and industrial civilization, worse human values that increase the likelihood of further global catastrophes, worse values being placed into artificial general intelligence, etc. Along with each percentage answer, please indicate your confidence level from 1 to 10. Note the numbered questions pertain to catastrophes that could collapse agriculture, while the lettered questions are on 10% global agricultural shortfalls.

Note: Confidence values are included but were not utilized in model.

**Table 7:** Relevant question from the survey and responses

3. a) What is your estimate of the reduction in the potential of humanity from catastrophes that reduce global agricultural output by 10% suddenly (e.g. regional nuclear war like between India and Pakistan, or a volcanic eruption like the one that caused the year without a summer in 1816) (if there had been no ALLFED)?		
Respondent	% Reduction	Confidence
1	0.03%	5
2	0.3%	5
3	20%	NP^7^
4	40%	4
5	0.1%	3
6	20%	5
7	0.05%	1
8	25%	3

^7^*NP* Not Provided

## Footnotes

1 This vulnerability can be addressed with distributed generation and microgrids (S. M [7, 65, 103, 136]), but these technologies are still far from ubiquitous.

2 Between 2015 and 2021 Open Philanthropy’s grantmaking to the “Potential Risks from Advanced Artificial Intelligence” cause area comes to a total of $156,488,525 USD, as compared to $92,634,721 USD granted to “Biosecurity and Pandemic Preparedness” [97].

3 One can change numbers in viewing mode to see how outputs change, but alterations will not save. If one wants to save a new version, one can make a copy of the model. Click View, visible to show arrows of relationships between cells. Drag mouse over cells to see comments. Click on the cell to show the equation.

4 On any given day, Ebay lists numerous used shortwave radio transmitter/receivers still in fully operational condition, some of them manufactured in the 1960s.

5 Ratios of means require manual changes in Guesstimate, which we note in all caps in the model.
